# Reovirus Activated Cell Death Pathways

**DOI:** 10.3390/cells11111757

**Published:** 2022-05-27

**Authors:** Carly DeAntoneo, Pranav Danthi, Siddharth Balachandran

**Affiliations:** 1 Blood Cell Development and Function Program, Fox Chase Cancer Center, Philadelphia, PA 19111, USA; carly.deantoneo@fccc.edu; 2 Molecular and Cellular Biology and Genetics, Drexel University, Philadelphia, PA 19102, USA; 3 Department of Biology, Indiana University, Bloomington, IN 47405, USA; pdanthi@indiana.edu

**Keywords:** reovirus, necroptosis, apoptosis, oncolysis, ZBP1, RIPK3, MLKL

## Abstract

Mammalian orthoreoviruses (ReoV) are non-enveloped viruses with segmented double-stranded RNA genomes. In humans, ReoV are generally considered non-pathogenic, although members of this family have been proven to cause mild gastroenteritis in young children and may contribute to the development of inflammatory conditions, including Celiac disease. Because of its low pathogenic potential and its ability to efficiently infect and kill transformed cells, the ReoV strain Type 3 Dearing (T3D) is clinical trials as an oncolytic agent. ReoV manifests its oncolytic effects in large part by infecting tumor cells and activating programmed cell death pathways (PCDs). It was previously believed that apoptosis was the dominant PCD pathway triggered by ReoV infection. However, new studies suggest that ReoV also activates other PCD pathways, such as autophagy, pyroptosis, and necroptosis. Necroptosis is a caspase-independent form of PCD reliant on receptor-interacting serine/threonine-protein kinase 3 (RIPK3) and its substrate, the pseudokinase mixed-lineage kinase domain-like protein (MLKL). As necroptosis is highly inflammatory, ReoV-induced necroptosis may contribute to the oncolytic potential of this virus, not only by promoting necrotic lysis of the infected cell, but also by inflaming the surrounding tumor microenvironment and provoking beneficial anti-tumor immune responses. In this review, we summarize our current understanding of the ReoV replication cycle, the known and potential mechanisms by which ReoV induces PCD, and discuss the consequences of non-apoptotic cell death—particularly necroptosis—to ReoV pathogenesis and oncolysis.

## 1. Introduction

Mammalian orthoreoviruses (ReoV) are grouped within *Reoviridae*, a family of non-enveloped RNA viruses with segmented double-stranded RNA (dsRNA) genomes. Reoviruses are ubiquitous and can infect a wide range of animals, including mice, pigs, sheep, bats, and birds. There are four mammalian ReoV serotypes, designated 1, 2, 3, and 4, which can be distinguished from each other by hemagglutination inhibition or neutralization techniques [1]. In adult humans, ReoV infections are largely asymptomatic. However, more recent evidence has linked these viruses to the development of chronic illnesses like Celiac disease [2]. Indeed, in neonatal animals, ReoV can have severe pathogenic consequences and is capable of inducing pneumonia, myocarditis, meningitis, and encephalitis [3,4,5,6,7]. 

ReoV was one of the first viruses successfully used for oncolytic therapy [8]. As an oncolytic virus, ReoV can hijack epidermal growth factor receptor (EGFR)- and rat sarcoma virus (Ras)-activating mutations to preferentially replicate in tumor cells [3]. ReoV can also take advantage of diminished type I interferon responsiveness within tumors to boost progeny virion production [9]. Finally, the ReoV genome encodes proteins capable of suppressing host innate immune pathways, which might otherwise impede viral replication [10]. Subsequent induction of programmed cell death (PCD) contributes to the destruction of tumor cells [9,11]. Such PCD can be augmented with other established chemotherapies, including gemcitabine or paclitaxel, and with immunotherapy to enhance eradication of tumor cells [12,13]. 

It was previously believed that the primary PCD pathway triggered by ReoV infection was apoptosis. However, new studies indicate that ReoV also activates necroptosis, a caspase-independent pathway of programmed necrotic cell death [14]. Although necroptosis is a potently effective standalone antiviral mechanism, it is also highly inflammatory [15,16,17]. Indeed, deploying necroptosis in tumors can reverse unresponsiveness to immunotherapy, and ReoV-induced necroptosis within the controlled context of the tumor microenvironment may be a significant contributor to its antitumor activity [18]. Additionally, ReoV may activate autophagy and pyroptosis, a form of cell death which combines characteristics of both apoptosis and necroptosis. Understanding how ReoV activates these additional PCD pathways is thus important for the effective utilization of the virus as an oncolytic agent. 

In this review, we summarize our current understanding of the ReoV replication cycle. We also discuss ReoV-activated PCD pathways, and outline both the known and unknown mechanisms of PCD triggered during ReoV infection. Finally, we consider the consequences of cell death, particularly inflammatory PCD, to ReoV pathogenesis and oncolysis. 

## 2. ReoV Structure, Genome, and Replication

**Figure 1:**
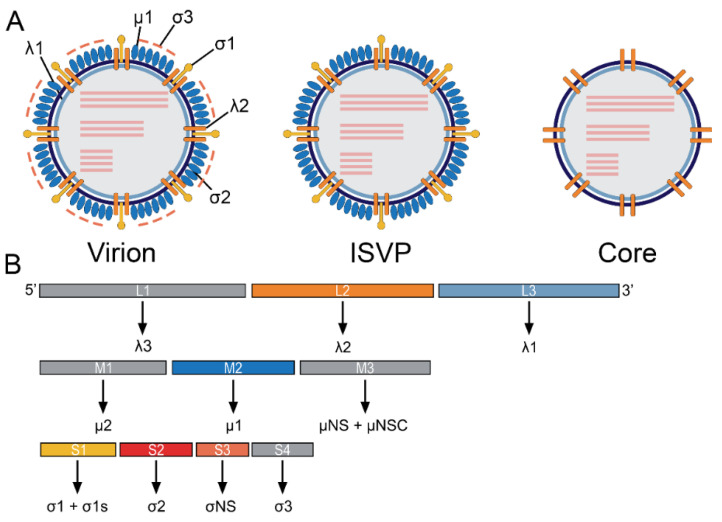
ReoV structure and genome organization. (**A**) Schematic depiction of the mature virion, containing all virally encoded proteins (**left**); the ISVP, a replication intermediate which has shed the σ3 protein (**middle**); and the viral core, lacking σ3, σ1, and µ1 (**right**). Viral proteins are indicated by black lines. Viral dsRNA genome segments are portrayed as pink lines. (**B**) ReoV genome comprises 10 dsRNA segments, depicted as rectangles. The proteins encoded by each segment are portrayed below.

ReoV is a non-enveloped RNA virus with 10 linear double-stranded genomic segments, ranging from ~1.1 to 3.5 kilo-base pairs in size. The ReoV virion comprises an ~80 nm icosahedral capsid with μ1-σ3 heterohexamers and up to 12 σ1 trimers (Figure 1A, left) [1,19]. Four main serotypes of mammalian ReoV have been identified. The primary divergence between these isolates lies within the RNA segment encoding σ1, the outer capsid protein responsible for attachment to host cells [20,21]. These serotypes are represented by prototype strains Type 1 Lang (T1L), Type 2 Jones (T2J), Type 3 Dearing (T3D), and Type 4 Ndelle (T4N) [1,22]. 

The viral genome encodes a total of 12 proteins (Figure 1B and Table 1). Viral proteins λ1, λ2, and σ2 form the viral core that protects the genomic dsRNA. The core is surrounded by the outer capsid comprised of σ1, σ3 and μ1, which also mediate entry into host cells. Following uptake of particles into the endosome, σ3 is proteolytically removed, exposing μ1 fragments µ1N and µ1C on viral particles. These fragments are further cleaved into δ and φ by endosomal proteases, which aid in release of the viral core into the cytoplasm [19,23]. Once inside of the cell, viral proteins such as σNS, µNS, and µ2 aid in the creation of viral factories, while the RNA-Dependent RNA polymerase (RdRp) λ3 acts to produce viral RNA strands [24,25,26,27]. 

**Figure 2:**
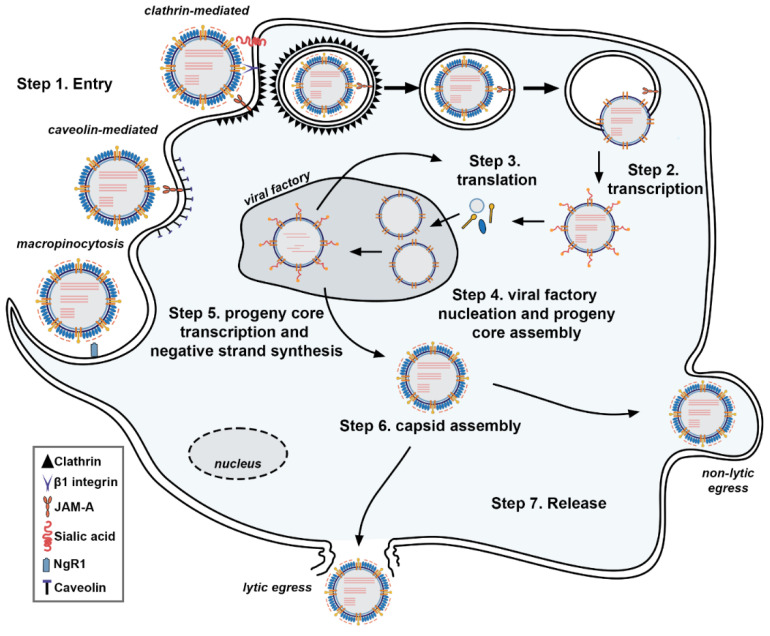
Reovirus Life Cycle. ReoV enters cells (Step 1) following binding of σ1 protein to host cell receptor, such as JAM-A and, in some cases, sialic acids. Clathrin-mediated endocytosis is the most common entry mechanism, although caveolin-mediated endocytosis and macropinocytosis is also possible. The virions are then shuttled within endosomes. Proteolysis degrades the outer σ3 protein and cleavage of the unveiled µ1C into fragments δ and φ, leading to formation of the ISVP. The µ1C cleavage fragments, along with µ1N, form pores within the endosomal membrane, depositing the viral core now lacking both µ 1 and σ1. Transcription by the RdRp λ3 occurs within the viral core underneath channels formed by λ2 (Step 2). Capping of mRNA is mediated by λ2. Viral mRNA is exported from the viral core and into the cytoplasm, where translation occurs (Step 3). Viral proteins mediate nucleation of viral factories, where progeny cores begin to self-assemble (Step 4). Negative strand synthesis occurs within progeny cores, forming nascent viral genomes (Step 5). Progeny core transcription occurs, and outer capsid proteins begin to assemble around progeny cores (Step 6). Finally, progeny virons leave the cell, either via lytic or non-lytic egress (Step 7).

The major steps of the ReoV life cycle are summarized in Figure 2. ReoV primarily infects humans and other mammals via the oral route, and replicates within cells of the intestinal epithelial tract [1]. Infection by the intranasal route has also been reported, with viral replication in airway epithelia leading to severe flu-like symptoms in susceptible hosts. In mouse models, some strains spread to the heart, causing myocarditis, or to the central nervous system (CNS), infecting neurons and inducing lethal encephalitis in newborn mice [4,5,6,7]. 

ReoV initiates infection by binding to sialylated glycans on the host cell, employing its outer capsid protein σ1 for this purpose. A-2,3, α-2,6, or α-2,8 linked sialylated glycans have all been reported to mediate or facilitate entry [28,29]. Glycan binding to one part of σ1 enables strong multivalent anchorage of the σ1 C-terminal head domain to junctional adhesion molecule A (JAM-A), an immunoglobulin superfamily receptor [30,31]. Interfering with σ1 binding to sialic acid or to JAM-A impedes ReoV infectivity [32,33]. ReoV also has the capacity to infect neurons, utilizing the neuron-specific Nogo receptor, NgR1, for entry into this cell type [5]. 

ReoV mainly uses clathrin-mediated endocytosis to enter cells, although it is also capable of engaging caveolin-dependent endocytosis and macropinocytosis to promote entry (Figure 2) [34,35,36]. During clathrin-dependent endocytosis, the ReoV λ2 protein interacts with the extracellular domains of host β1 integrins, initiating cytoskeletal rearrangements which permit entry into the cell [34,37]. The virion then undergoes proteolytic disassembly, utilizing cathepsin family proteases within the endosome for this purpose [38]. Key steps in viral capsid disassembly involve proteolytic degradation of viral σ3 and cleavage of μ1C. In its unmodified state, σ3 protects μ1C from cleavage, so it is not until σ3 is degraded by cellular proteases that μ1C is capable of being cleaved into two fragments: δ and φ. The μ1C cleavage fragments undergo conformational changes, exposing hydrophobic residues which, together with myristoylated µ1N, insert into the endosomal membrane, puncturing the endosome and releasing the core into the cytoplasm for transcription [39]. The transcriptionally active virion core (Figure 1A, right) is then released into the cytosol [19]. Of note, activity of the cellular kinase Src is necessary for proper targeting of the virion to an endocytic compartment for eventual disassembly [35,40]. 

Caveolin-mediated endocytosis is most likely to occur within the intestinal lumen, where secreted serine proteases can digest σ3 to produce extracellular infectious subviral particles (ISVPs) (Figure 1A, middle) which are then internalized by caveolin-dependent mechanisms [38]. Macropinocytosis permits entry of ReoV into neurons and is driven by cytoskeletal rearrangement and phosphoinositide 3-kinase (PI3K) activity [36]. In each case, the downstream events following virion entry into the cell are similar to those which occur after clathrin-dependent endocytosis.

ReoV replication takes place within cytoplasmic viral factories (sometimes called viral inclusion bodies) composed of remodeled ER membranes and nucleated by ReoV μ2, σNS, and μNS proteins [24,25,26]. Early viral RNA transcription occurs soon after core delivery into the cytoplasm and the removal of μ1. Changes in λ2 structure form a channel, allowing nucleotide entry and export of viral mRNA from the viral core into the cytoplasm [41,42].

λ3, the viral RNA-dependent RNA polymerase, does not require an RNA primer to initiate transcription. Instead, λ3 is recruited to the parent template by a conserved sequence present at the 5′ end of the template strand. Messenger RNAs (mRNAs) are synthesized from the negative-sense strand of genomic dsRNAs and capped during transit through the λ2 channel. λ2 possesses a guanyltransferase and two methyltransferase domains needed for capping, while the NTPase and helicase activities of µ2 and λ1 are likely involved in unwinding genomic dsRNAs prior to transcription and capping [43,44,45]. Once capped, these mRNAs are exported from the viral core through the λ2 channel and deposited into the cytoplasm for translation into viral proteins by host ribosomes. 

Translation of viral mRNAs is driven by ribosomal scanning of ReoV mRNAs to identify the initiator codon (AUG), with σ3 serving to stimulate translation by interacting with ribosomes. ReoV also utilizes alternative open reading frames for synthesis of some proteins, such as σ1S and μNS [46]. Once proteins necessary for the inner viral capsid are synthesized, self-assembly of new progeny cores occurs. mRNA is packaged within these new cores, and replication takes place inside the core to generate nascent dsRNA genomes [47]. This stage of replication can be blocked by guanidine hydrochloride (GuHCl), which inhibits transcription of negative sense RNAs within cores and prevents genome amplification [43,48]. dsRNA containing progeny cores can also synthesize additional mRNAs called “secondary transcripts.” Progeny virion assembly occurs once outer capsid proteins are synthesized. As with formation of the viral core, much of the outer components of the virion self-assemble. Host Hsp70/90 chaperones are required for proper folding of σ1, and the T-complex protein ring complex (TRiC) chaperonin is necessary for folding of σ3 during outer core assembly [49]. 

ReoV progeny virions release occurs predominantly at the apical surface of polarized cells [44,45]. ReoV can employ either lytic or non-lytic mechanisms for egress, depending on the type of cell infected. Non-lytic egress can involve use of modified host lysosomes; during replication, viral factories can become internalized into lysosomes, which then ferry progeny virions to the plasma membrane for exit into the extracellular space [50]. Lytic egress is achieved by disruption of the cell membrane integrity, although the mechanisms by which ReoV triggers lysis to promote virion release are not well defined. Moreover, whether particular PCD pathways promote or hinder virion release, and the ways in which PCD impacts downstream anti-ReoV immune responses, are unclear. Illuminating how ReoV activates PCD pathways, how these pathways contribute to the innate and adaptive immune responses to ReoV infection, and their impact on pathogenesis of this virus are important areas of investigation. The next two sections summarize what is known about innate sensing pathways activated during ReoV infection, and the PCD pathways triggered by this virus. 

## 3. ReoV and Innate Immunity

The dsRNA genome of ReoV can activate innate immune RNA sensors, often culminating in regulated cell death. These RNA sensors include RIG-I-like receptor (RLRs) family members retinoic acid-inducible gene-I (RIG-I) and melanoma differentiation-associated 5 (MDA5) [61,66]. RLR activation stimulates mitochondrial antiviral-signaling (MAVS) protein on the outer mitochondrial membrane, which then engages inhibitor of nuclear factor kappa-B kinase (IKK)-α, IKK-β, IKK-ε, and Tank-binding kinase 1 (TBK1) kinases. This results in activation of transcription factors such as nuclear factor kappa-light-chain-enhancer of activated B cells (NF-κB) and IFN regulator factor 3 (IRF-3), and cumulates in production of type I interferons (IFN-I) [67,68]. This IFN response has several important roles in restricting virus replication and promoting adaptive immune responses, such as promoting antigen presentation and driving immune cell recruitment. Essential to the antiviral effects of IFN-I is the induction of ISGs, such as *Trail* and *Puma*, which encode modulators of PCD [62,69,70]. In mouse fibroblasts lacking either RIG-I or MDA5, the ReoV-elicited IFN-I response is greatly reduced and PCD is dampened [61]. Similarly, ablating MAVS leads to near-complete loss of an IFN-I response following ReoV infection [70]. 

Unlike the case with RLRs, the importance of TLR3 to controlling ReoV infections is still unclear. One study suggests TLR3 is necessary for the immune response against ReoV, as knockdown of TLR3 with siRNA reduces the response both in cellulo and in tumor xenograft models [63]. However, during ReoV infections of the CNS, the TLR3-mediated innate response does not appear to be critical for virus clearance [64,65]. Furthermore, the T1L strain, unlike the T3D strain, is unaffected by TLR3 deficiency within intestinal cells, suggesting that strain-specific differences may also dictate TLR3-dependent innate immune responses [71].

Apart from RLRs and TLR3, other RNA-sensing host proteins, such as DexH-Box Helicase 9 (DHX9), are activated within infected dendritic cells [72]. DHX9 possesses a HelicC-HA2-DUF domain, which can interact with the caspase recruitment domain (CARD) in MAVS, leading to IRF3/NF-κB activation. Similarly, DHX36 and DEAD-Box helicase 1 (DDX1) and DDX21 may also sense ReoV dsRNA and stimulate IFN-I production [72]. DHX33 binds ReoV RNA and can activate the NLRP3 inflammasome, potentially linking ReoV infection to activation of pyroptosis. DDX41 may also sense ReoV dsRNAs and activate IFN-I production through STING [73,74]. Knocking down any of these helicases, or their downstream effectors, reduces ReoV-induced activation of IRF3 and NF-κB, and limits the IFN-I response in cellulo [73,75,76]. The role of these helicases in control of ReoV spread in vivo is unknown. 

Of note, the E3 ligase tripartite motif-containing protein 29 (TRIM29) may promote ReoV replication by reducing IFN-I responses. In dendritic cells and macrophages, TRIM29 mediates K11-linked polyubiquitination of MAVS, thus inhibiting IRF3 activation [77]. Additionally, TRIM29 degrades the NF-κB essential modulator (NEMO), limiting NF-κB activation and reducing IFN-I signaling [78]. Ablating TRIM29 expression in mice leads to significant reduction in ReoV titers and an increase in IFN-I signaling, demonstrating its importance to ReoV control in vivo [77,78].

## 4. ReoV-Induced Non-Necroptotic Cell Death Pathways

### 4.1. Apoptosis

Apoptosis is the best characterized PCD mechanism activated during ReoV infections. Distinct morphological features are induced during apoptosis, including cell shrinkage, membrane blebbing, chromatin condensation, and eventual collapse of the cell into discrete membrane-bound apoptotic bodies. These apoptotic bodies are typically phagocytosed by surrounding cells, without leakage of potentially inflammatory intracellular contents, and apoptosis is thus not typically associated with a strong inflammatory response [79]. 

Both cell-intrinsic and -extrinsic pathways can trigger apoptosis. Intrinsic, or mitochondrion-dependent, apoptosis occurs when the inner mitochondrial membrane is disrupted, leading to release of cytochrome c, as well as second mitochondrial activator of caspases (SMAC)/Diablo and Htr/Omi, into the cytosol [80]. Cytochrome c then interacts with Apaf-1 and procaspase-9 to form the apoptosome, which then leads to activation of caspase-3 and subsequent endonuclease and protease activation. Chromosomal DNA and cellular proteins are then cleaved, degraded, and packaged into discrete membrane-bound vesicles, culminating in apoptotic cell death [81]. SMAC/Diablo and Htr/Omi inactivate X-linked inhibitor of apoptosis (XIAP), promoting effector caspase activation [80]. Under normal conditions, mitochondrial permeability is controlled by B cell lymphoma 2 (Bcl-2) family proteins, which prevent proapoptotic Bax and Bak proteins from activating apoptosis. When Bcl-2 is inhibited, Bax and Bak become active, disrupt the mitochondrial membrane, and induce apoptosis [81].

**Figure 3:**
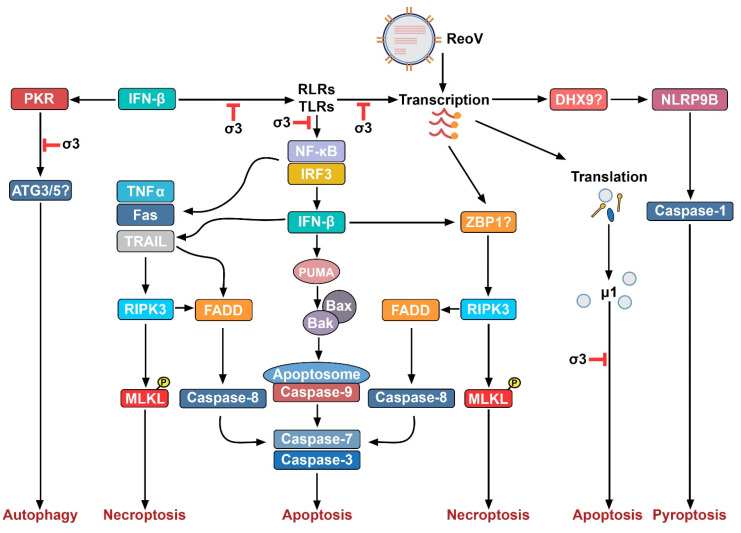
Programmed Cell Death Pathways activated by ReoV. This figure illustrates four potential programmed cell death pathways activated by ReoV infection. Potential dsRNA sensors in these pathways are indicated by question marks.

Extrinsic apoptosis is activated by death ligands, such as tumor necrosis factor α (TNFα), TNF-related apoptosis inducing ligand (TRAIL), and Fas Ligand (FasL). These ligands bind their cognate receptors on the cell surface and initiate formation of a complex containing proapoptotic adaptor molecules such as Fas associated death domain (FADD) or TNF receptor type 1-associated death domain protein (TRADD). These in turn associate with procaspase-8, which then undergoes auto-activation and cleaves executioner caspases, such as caspase-3, resulting in apoptosis [81,82,83]. Caspase-8 can also cleave and activate Bid, which translocates to the mitochondria and potentiates Bax-driven apoptotic signaling, linking extrinsic and intrinsic pathways of apoptosis. Some strains of ReoV also promote upregulation of proapoptotic genes (e.g., those encoding FasL and TRAIL), amplifying apoptosis [75,84]. Both intrinsic and extrinsic apoptosis triggered during ReoV infection are summarized in Figure 3. 

ReoV can activate apoptotic pathways within murine and human cell lines in culture, and in the murine intestine, heart, and CNS in vivo [15,85,86]. ReoV nucleic acids can trigger both intrinsic and extrinsic pathways of apoptosis by directly activating host RNA-sensing proteins, such as RLRs, leading to activation of the transcription factors NF-κB and/or IRF3, and induction of proapoptotic targets such as Noxa and PUMA. Noxa and PUMA then inhibit Bcl-2, activating Bax/Bak and apoptosis [62,87]. NF-κB and IRF3 also cooperate to induce expression of IFN-Is, stimulating induction of proapoptotic ISG products (such as TRAIL) that, in turn, can activate apoptosis [80]. During ReoV infections of neurons, Bid becomes proteolytically activated and amplifies apoptosis [76]. Additionally, the ReoV protein μ1 can directly provoke apoptosis, particularly during viral entry [57,88]. This pathway may be independent of Bax/Bak activation, as μ1 protein, via its φ domain, can form pores in mitochondria [89]. To prevent this, the ReoV σ3 protein typically binds μ1, limiting the capacity of μ1 to form pores and induce apoptosis [57]. 

Apoptosis is considered an essential host defense mechanism against viral infection because it not only eliminates infected cells (thus preventing them from becoming virus factories), but also galvanizes antiviral adaptive immune responses by supplying virus antigen for cross-presentation to antiviral T cells. Indeed, ReoV strains which induce more apoptosis in culture are typically cleared more quickly in vivo, compared to those strains less able to induce apoptosis [90]. However, there are instances where the virus may use apoptosis to aid its own release and dissemination. For example, although ReoV can activate Bid to amplify apoptosis, ReoV infection of Bid-deficient mice results in decreased virus yield and increased overall survival rates, compared to similarly infected control animals [91]. Furthermore, ReoV mutants in which the proapoptotic function of µ1 and σ1 is selectively abolished display significantly reduced virulence [92,93,94]. 

The deleterious effects of apoptosis to the host may be cell-type specific, as when apoptosis signaling is disabled (e.g., in caspase-3-deficient settings), the most significant drop in virus replication and pathology is observed in CNS tissues, but not in the intestines, liver, or heart [92,93,95,96]. Similarly, ReoV-triggered apoptosis induced lethal encephalitis and tissue damage in the brains of wild-type, but not caspase-3-deficient, mice [97]. Interestingly, the negative consequences of ReoV-activated apoptosis to the infected host do not appear to be linked to the extent of viral replication, as apoptosis activation is not dependent on robust viral replication in the CNS [97]. Thus, while apoptosis may be important for limiting ReoV spread, additional research is necessary to dissect the possible cell type-specific roles of apoptosis in promoting versus preventing anti-ReoV host defense.

### 4.2. Autophagy

Autophagy is a biochemically programmed intracellular degradation process, during which cellular contents are encapsulated and degraded within membrane-bound cytoplasmic vesicles called autophagosomes. Although autophagy is generally thought of as a “housekeeping” process necessary for cellular homeostasis during periods of nutrient deprivation, it can also directly target and aid in the disposal of intracellular pathogens [98]. During viral infections, Beclin-1, an autophagy protein required for nucleating autophagosomes, becomes activated when suppressive Beclin-1:Bcl-2 interactions are lost. This allows Beclin-1 to instead associate with Myeloid differentiation primary response 88 (MYD88) and TRIF, triggering autophagy. These autophagosomes then can engulf and sequester viral components, delivering them to lysosomes for degradation [99]. As is the case with interactions between ReoV and the apoptosis machinery, reoviruses have evolved ways to evade or hijack the autophagy machinery for its own benefit. 

Much of our insight into how ReoV activates autophagy comes from work on avian ReoV strains (e.g., GX/2010/1), which promote autophagy by upregulating 1A/1B light chain 3B (LC3) and downregulating the mammalian target of rapamycin (mTOR), a negative regulator of autophagic signaling [100] (Figure 3). Avian ReoV strains utilize autophagic signaling to increase their own reproduction and dissemination by mechanisms that are as-yet undetermined [85,101]. In agreement with this observation, ablating PI3K or autophagy related lipidation proteins 3 and 5 (ATG3/5) in ReoV infected cancer cells reduces viral replication and the amount of progeny virions produced [86,102]. Together, these results suggest that both avian and mammalian ReoV strains co-opt autophagy to promote their own replication and dissemination, although the method through which this is achieved is still not fully clear. 

Notably, autophagy is closely linked to the activation of apoptosis. For example, Beclin-1 interacts with and inhibits BCL-2 function, promoting activation of the intrinsic pathway of apoptosis [95]. During avian ReoV infections, inhibiting autophagy also reduces apoptosis and increases survival of the host cell [92]. Finally, in human clinical trials, mammalian ReoV has been found to induce both autophagy and apoptosis, particularly in cancer subtypes with mutations which increase autophagic flux beyond homeostatic levels [102,103].

### 4.3. Pyroptosis

Pyroptosis is a form of lytic programmed cell death activated by inflammasomes in response to both microbial infections and environmental insults. Inflammasomes are multi-protein complexes which become activated following sensing of pathogen-associated molecular patterns (PAMPs) and damage-associated molecular patterns (DAMPs) to trigger caspase-dependent signaling cascades [93,94,104]. Inflammasome-activated caspases cleave cytosolic gasdermins, producing a N-terminal domain that oligomerizes and forms pores in the cell membrane, triggering membrane rupture and cell death. The cellular contents dumped into the intracellular space can then act as DAMPs and PAMPs to surrounding cells, amplifying inflammation. Additionally, caspases cleave and activate the inflammatory cytokines IL-18 and IL-1β, which are released by pyroptotic cells [93,94,104]. 

There are three current models for pyroptosis activation: the canonical pathway, the noncanonical pathway, and a caspase-3/8-dependent pathway [96,105]. The canonical pathway involves sensing of DAMPs and PAMPs by cytosolic sensors, such as NOD-like receptors and pyrin-domain containing family members 1 and 3 (NLRP1/3). Absent in melanoma 2 (AIM2) protein and IFN-γ inducible protein 16 (IFI16), they can also directly sense PAMPs to trigger canonical pyroptosis. Once these canonical sensors are activated, they trigger assembly of the apoptosis-associated speck-like protein (ASC), causing its CARD to bind the CARD on pro-caspase 1, thus forming the inflammasome [96,105]. In the non-canonical pathway, pro-caspase 4/5 (pro-caspase 11 in mice) can directly sense lipopolysaccharides produced by invading gram-negative bacteria, triggering caspase maturation and direct cleavage of gasdermin D (GSDMD). Finally, the caspase-3/8 dependent pathway involves TGF-β-activated kinase 1 (TAK1) induction of caspase-8, leading to cleavage of GSDMD [96]. 

Little is known about how ReoV activates inflammasomes and triggers pyroptosis. Other RNA viruses, such as encephalomyocarditis virus (EMCV), vesicular stomatitis virus (VSV), and IAV (influenza A virus), however, have been proven to activate the NLRP3 inflammasome, and DXH33 has been suggested to link ReoV replication to NLRP3 activation during ReoV infection [75,84]. Additionally, rotaviruses, which are closely related to ReoV, can also induce pyroptosis, but may do so via the NLRP9B inflammasome. During rotavirus infection, the Dhx9 RNA helicase senses rotavirus dsRNA, triggering NLRP9B inflammasome complex formation, activation of caspase-1, and pyroptosis [106] (Figure 3). Whether or not mammalian ReoV triggers a similar NLRP9b-dependent pathway of pyroptosis warrants further investigation. 

## 5. ReoV and Necroptosis

Until recently, necrotic death was considered the unprogrammed consequence of cellular injury following strong external insults, such as exposure to toxins or to mechanical trauma. Over the past decade, a programmed pathway of necrosis reliant on the kinase RIPK3 and its substrate MLKL has been described [107,108] (Figure 3). Necroptosis may be induced by a variety of mechanisms but is always mediated by RIPK3. When necroptosis is activated by the TNF family of death receptors (DRs), the kinase RIPK1 mediates recruitment and activation of RIPK3 via the receptor-interacting protein (RIP) homotypic interaction (RHIM) motifs found in both proteins [109,110]. In other pathways, such as those initiated by TRIF and ZBP1, RIPK1 is not essential for RIPK3 activation. Instead, RIPK3 can interact directly with TRIF or ZBP1, again via a RHIM-RHIM association. In either case, once RHIM interactions occur, RIPK3 oligomerizes and autophosphorylates. Following this, RIPK3 phosphorylates MLKL, inducing a major conformational change that releases MLKL’s N-terminal 4-helix bundle, which possesses strong affinity for a variety of phosphatidylinositol moieties found on cellular membranes. Phosphorylated MLKL then associates with these moieties, perforates the cell membrane, and ruptures the cell [109,110].

Necroptosis is distinct from apoptosis in important ways. Firstly, it is independent of caspase activity, and does not result in DNA fragmentation; second, its morphological features involve swelling and plasma membrane rupture; finally, whereas apoptosis is typically not inflammatory, necroptosis can provoke a strong inflammatory response by releasing DAMPs and alarmins from the ruptured cell into the extracellular space [15].

ReoV was first found to activate necroptosis in T3D-infected L929 cells, which displayed rampant cell death even when treated with caspase inhibitors [14]. Such cell death was accompanied by membrane rupture and inhibited by necrostatins, demonstrating that it was necroptotic in nature. In a later study, it was found that IFN-β production following the detection of incoming viral genomic RNA is required, but not sufficient, for eliciting necroptosis. In addition to IFN-β expression, de novo synthesis of viral dsRNA was also required for necroptosis induction [70]. The requirement for both IFN-β and de novo synthesized dsRNA in activating necroptosis was demonstrated by indicating that (1) exposing ReoV RNA to calf intestinal phosphatase (CIP) treatment (which removes 5’ phosphates from RNA ends and diminishes their capacity to activate RIG-I), or ablating expression of RLR-signaling components in cells, reduced death of T3D infected cells; and (2) selectively blocking second-round (i.e., de novo) viral genome synthesis (with GuHCl) also prevented necroptosis [70]. Together, these results suggest that incoming genomic RNA is detected by RLRs in the cytoplasm of the infected cell, which then signal via the adaptor protein MAVS to produce IFN-β and other IFN-Is. As necroptosis is blocked under conditions where IFN-β is still produced, but new viral dsRNAs are not synthesized (i.e., in GuHCl-treated cells), these studies further indicate that, except for IFN-I signaling, newly synthesized viral dsRNA is also required for the initiation of necroptosis, likely because these dsRNAs serve as necroptosis-activating ligands for an as-yet unidentified ISG product. 

Given the requirement for newly synthesized dsRNA species in activating necroptosis, subsequent studies have evaluated the role of viral factors that increase dsRNA synthesis or factors which control the exposure of viral dsRNA to host-sensing proteins in influencing necroptosis outcomes. Knockdown of ReoV μ1 protein accelerated necroptosis following infection without affecting apoptosis [48]. Knockdown of μ1 also increased accrual of progeny dsRNA and viral protein synthesis. These data highlight a new function for μ1 in controlling the levels of viral gene products (both RNA and protein) in infected cells, and suggest that viral replication products produced later in infection are detected by the host innate immune machinery to elicit necroptotic cell death. In related studies, it was found that ablating expression of the ReoV σ3 protein (which binds dsRNA) enhanced necroptosis [111]. σ3 ablation did not much impact ReoV RNA synthesis; instead, elevated necroptosis following σ3 knockdown was accompanied by an increase in RLR-driven IFN-β production, and therefore likely the result of enhanced type IFN signaling and consequent induction of the ISG-encoded necroptosis-initiating sensor protein(s). Although ectopic expression of σ3 was sufficient to block IFN expression in infected cells, the ability of σ3 protein to bind dsRNA surprisingly did not impact its ability to dampen IFN production. In fact, infection with a ReoV mutant carrying an inactivating alteration in the dsRNA binding domain of σ3 did not result in either enhanced IFN production or in increased necroptosis [111]. Thus, σ3 limits the production of IFN—and consequent necroptosis—by a mechanism which appears to be independent of its ability to bind and sequester dsRNA from RLRs. How σ3 might prevent IFN production independent of its ability to bind and sequester dsRNA warrants further exploration. 

The mechanism by which ReoV infections initiate necroptosis signaling is still unknown. One suggested possibility involves the downregulation of cIAP1, an E3 ubiquitin ligase whose levels are known to be suppressed during ReoV infection [112]. As cIAP family E3 ligases (cIAP1 and cIAP2) can polyubiquinate RIPK1 and RIPK3 and inhibit their kinase activity, downregulating cIAP1 during ReoV infection may lead to more active RIPK1/3 kinases, and thus to more necroptosis [113]. However, modulation of necroptosis outcomes at the level of cIAP1 does not explain how necroptosis is initiated in the first place, or why de novo dsRNA synthesis is required for necroptosis activation [103]. 

In this regard, ZBP1, a host protein capable of sensing Z-form (left-handed) dsRNA species is a strong candidate for upstream initiator of necroptosis signaling following ReoV infection. ZBP1 is encoded by an ISG, binds dsRNA (albeit in the Z-conformation), and triggers necroptosis in other settings, fulfilling the criteria for the “missing link” necroptosis initiator during ReoV infections (Figure 3) [114,115]. Indeed, publicly available RNA-seq data indicate that the mRNA encoding ZBP1 is highly upregulated in ReoV infected cells, and blocking IFN-I receptor signaling during ReoV infection results in significantly decreased levels of *Zbp1* mRNA [1,52,78,116]. 

Also unclear is the relevance of necroptosis to ReoV clearance and pathogenesis, and the cell types in which necroptosis occurs during ReoV infections in vivo. Bone marrow-derived macrophages (BMDMs) and L929 fibroblasts undergo necroptosis within 1-2 days post-infection in culture [6,52,78]. Whether ReoV induces necroptosis within cell types of the gut, CNS, and heart remains unknown. Additionally, whether such necroptosis leads to tissue damage and disease during ReoV infection—or potentially to enhanced virus clearance and reduced pathology—is also unknown. Indeed, necroptosis following infection with other viruses (such as IAV) may act as a double-edged sword, facilitating viral clearance when well-controlled but also inducing harmful hyper-inflammation when unchecked [15,117]. Whether necroptosis is protective or deleterious during ReoV infection warrants consideration. In this regard, loss of RIPK3-enhanced ReoV progeny virion output, indicative of a potentially protective role for necroptosis in ReoV clearance [70,118].

## 6. Implications for ReoV Oncolysis

Pioneering work from Donald Cox and Patrick Lee demonstrated that ReoV has strong potential as an oncolytic virus [10,119]. ReoV preferentially replicates in transformed cells, and at least three mechanisms have been proposed to explain this phenomenon. First, tumor-specific mutations, particularly activating epidermal growth factor receptor (EGFR) and Ras mutations, appear to predispose cells to ReoV replication and cell death [9]. They may do so by inhibiting the antiviral activity of PKR, permitting efficient ReoV translation [118]. Additionally, oncogenic Ras mutants can enhance ReoV spread by inhibiting RIG-I signaling, further dampening the innate immune response against ReoV within these cells. Ras mutations are common in many cancers; in multiple melanoma alone, over 50% of tumors carry N-Ras or Kirsten rat sarcoma virus (K-Ras) mutations, while over 45% of colorectal cancers carry similar alterations [100,103]. Mechanistically, Ras mutants can lower RIG-I protein levels by impairing translation of Ddx58 mRNA (which encodes RIG-I), achieved via active PI3K and Mitogen-activated protein kinase/extracellular signal-regulated kinase (MEK/ERK) signaling [9,120]. 

Mutations in other oncogenic pathways (e.g., PI3K/AKT, mTOR, and NF-κB) also sensitize cells to ReoV oncolysis, suggesting that a general increase in the proliferative capacity of a cell as it undergoes neoplastic transformation facilitates ReoV replication [62,120]. This is expected, as the increased availability of anabolites and other raw materials accompanying proliferation of tumorigenic cells will also boost virus replication rates in these cells. Secondly, most transformed cells deactivate essential innate-immune antiviral pathways during transformation, most notably pathways involved in IFN-I production and signaling; these tumor-specific defects render transformed cells more permissive to ReoV. Thirdly, ReoV itself suppresses innate immune pathways, for example by deploying σ3 to prevent PKR activation, which also leads to unimpeded translation of viral mRNAs and increased production of progeny virions [10]. 

ReoV as an oncolytic virus has been proven to boost the antitumor potential of standard chemotherapeutics [24]. As Pelareorep or Reolysin, ReoV strain T3D has been evaluated as an oncolytic virus in numerous phase I and II studies [8,12]. Genetic modifications to the virus have enhanced the oncolytic efficacy of ReoV [121]. For example, the T3V1 and T3V2 variants of T3D, which have modifications in their λ2 and σ1 proteins, manifest increased replication kinetics, and display reduced toxicity in infected mice compared to the wild-type T3D strain [122]. In most cases, ReoV was combined with established therapies, such as immune checkpoint blockade (ICB) antibodies or platinum-based compounds, in treatment of numerous cancer types, including ovarian, colorectal, head and neck, lung, breast, and skin cancer. The goal in these studies involved using ReoV to stimulate the immune response in patients undergoing conventional therapeutic regimens. For example, in one trial involving FOLFIRI/bevacizumab + Pelareorep, patients displayed enhanced dendritic cell maturation compared to those receiving FOLFIRI/bevacizumab alone. Other trials saw significant increases in CD4+ and CD8+ T cells, as well as upregulation of cytokines involved in immune cell recruitment, when ReoV was incorporated into the treatment regimen [123,124]. While the results from most of these trials are still pending, the available data have demonstrated that ReoV is a generally safe and well-tolerated agent with significant efficacy, both as a monotherapy and as an immune adjuvant in a range of tumor types. 

The role of PCD in ReoV induced oncolysis is not well defined. Much research stresses the potential role of so-called “immunogenic apoptosis,” particularly when combined with treatments such as radiotherapy, in inducing cancer cell death [3,19]. Such immunogenic cell death has the potential to reawaken the tumor microenvironment (TME) by triggering the release of DAMPS and PAMPs from infected cells [11]. Importantly, activation of other PCD pathways has been observed during oncolytic ReoV infection. For example, autophagy is upregulated in ReoV-infected melanoma cells, leading to the release of viral progeny, infection of neighboring cells, and eventual cell death [102,125]. Additionally, pyroptosis may play a similar role in promoting an immunogenic TME and enhancing immunotherapeutic outcomes, given than programmed cell death protein 1 (PD-1) antibodies manifest improved efficacy when pyroptosis is triggered in tumors [126]. 

Necroptosis is a powerful method of inflaming the tumor microenvironment and rekindling immune responses in otherwise “cold” tumors. In pre-clinical models, activating necroptosis in tumors (by adenoviral delivery of activated RIPK3, for example) triggers tumor cell necroptosis and antitumor immunity, an effect which synergizes with anti-PD-1 antibodies and other ICB agents [18]. Similarly, delivery of mRNA-encoding MLKL halted tumor growth in mouse melanoma and colon carcinoma models, particularly in conjunction with ICB agents. MLKL transfection also induced a strong CD4+ and CD8+ T cell response, mediated by IFN-I signaling and Batf3-dependent dendritic cells [116]. These studies indicate that ReoV-triggered necroptosis, either in tumor cells, or in cells of the TME, may contribute to its oncolytic potential. 

## 7. Future Perspectives

We have outlined three areas related to ReoV activation of PCD pathways that warrant further investigation. First, at the mechanistic level, the method by which ReoV activates apoptosis, autophagy, pyroptosis, or necroptosis is still not well defined. For example, the relative contributions of cell-extrinsic versus intrinsic apoptosis pathways to PCD following ReoV infection are unknown, as are the roles of individual members of the TNFR superfamily of proteins. How (or if) mammalian ReoV strains trigger autophagy and pyroptosis in relevant cell types—and the mechanisms involved—merit further investigation. Additionally, whether ZBP1 is the upstream sensor of ReoV in the necroptosis pathway of PCD remains to be clarified, and if ZBP1 is indeed the necroptosis-activating sensor of ReoV infections, then whether ReoV produces Z-RNAs capable of activating ZBP1 becomes an important question. 

Second, the importance of PCD to ReoV clearance versus pathogenesis is unresolved. For example, inhibition of apoptosis in CNS tissues leads to a significant drop in replication, but a similar trend has not been observed in the intestines, heart, liver, or other tissues from ReoV-infected mice [92,93,95,96]. Conversely, inhibition of autophagy during infection with avian ReoV was associated with increased progeny in chicken tissues [85,86,101,102]. Whether autophagy has a similar pro-viral role during mammalian ReoV infections, and the cell types in which this autophagy is important for ReoV replication, remains undefined. Also unknown is the potential function of pyroptosis or necroptosis in ReoV replication and pathology. 

Finally, we suggest that engineering ReoV strains to maximize their necroptosis-inducing potential will boost the therapeutic efficacy of the virus as an adjuvant for cancer immunotherapies. As a highly immunogenic cell death mechanism, necroptosis can synergize with immune-checkpoint blockade therapies, such as anti-PD-1 antibodies [18]. Localized activation of necroptosis induces strong immune cell responses, directing the host response engendered by ICB therapeutics to the tumor mass and creating a TME that is more responsive to treatment [116]. Thus, ReoV strains tailored to potentiate necroptosis will represent next-generation oncolytics with strong clinical potential.

## Figures and Tables

**Table 1:** ReoV proteins and their known functions. This table lists all proteins encoded by the ReoV genome, the segment which encodes each protein, the known functions of the proteins, and their location in the virion.

Encoded Protein	Genome Segment	Role in Viral Life Cycle	Role in the Immune Response	Location	Reference
λ3	L1	RNA-dependent RNA polymerase	Unknown	inner capsid	[27]
λ2	L2	Capping (methyltransferase and guanylyltransferase activity) Forms interactions with β1 integrin on host cell Forms a channel for the export of viral RNA	Unknown	inner capsid	[41,51]
λ1	L3	Possible helicase/NTPase Forms the viral core	Unknown	inner capsid	[52]
μ2	M1	RNA binding NTPase RNA triphosphatase Associates with host microtubules to aid in viral factory formation	Inhibits interferon signaling	inner capsid	[24,53,54,55]
μ1 (cleaved into μ1C and μ1N)	M2	Forms pores in endosomes	Induces apoptosis	outer capsid	[56,57]
μNS + μNSC	M3	Forms viral factories Provides scaffolding for progeny core assembly	Imhibits IRF3 signaling	non-structural	[26,55]
σ1 + σ1s	S1	σ1 binds to host cell receptor such as JAM-A Glycosidase	σ1 binds host cell σ1s can induce cell cycle arrest	σ1 = outer capsid σ1s = non-structural	[55,58,59,60]
σ2	S2	Interacts with λ1 to form the viral core dsRNA binding	Unknown	inner capsid	[28,45,61]
σNS	S4	RNA binding Viral factory formation May be involved in genome packaging	Unknown	non-structrual	[31,62,63]
σ3	S3	dsRNA binding May mediate binding to NgR1	Blocks PKR Blocks RLR signaling Binds µ1 to attenuate apoptosis	outer capsid	[37,64,65]

## Data Availability

Not applicable.
